# Mitochondrial Lysyl-tRNA Synthetase Independent Import of tRNA Lysine into Yeast Mitochondria

**DOI:** 10.1371/journal.pone.0035321

**Published:** 2012-04-23

**Authors:** Naresh Babu V. Sepuri, Madhavi Gorla, Michael P. King

**Affiliations:** 1 Department of Biochemistry, School of Life Sciences, University of Hyderabad, Hyderabad, Andhra Pradesh, India; 2 Department of Biochemistry and Molecular Pharmacology, Thomas Jefferson University, Philadelphia, Pennsylvania, United States of America; Max-Planck-Institute for Terrestrial Microbiology, Germany

## Abstract

Aminoacyl tRNA synthetases play a central role in protein synthesis by charging tRNAs with amino acids. Yeast mitochondrial lysyl tRNA synthetase (Msk1), in addition to the aminoacylation of mitochondrial tRNA, also functions as a chaperone to facilitate the import of cytosolic lysyl tRNA. In this report, we show that human mitochondrial Kars (lysyl tRNA synthetase) can complement the growth defect associated with the loss of yeast Msk1 and can additionally facilitate the *in vitro* import of tRNA into mitochondria. Surprisingly, the import of lysyl tRNA can occur independent of Msk1 *in vivo*. This suggests that an alternative mechanism is present for the import of lysyl tRNA in yeast.

## Introduction

Aminoacyl-tRNA synthetases are a heterogeneous family of enzymes responsible for aminoacylating tRNAs with the appropriate amino acids. The budding yeast, *Saccharomyces cerevisiae*, contains two sets of lysyl-tRNA synthetases (Msk1 and Krs1) that are encoded by nuclear DNA. Krs1 participates in cytoplasmic protein synthesis while Msk1 in mitochondrial protein synthesis. Despite the structures of the Msk1 and Krs1 being different, functionally they are very similar. The yeast Msk1 has a bacterial ancestry, whereas the Krs1 represents the ancestral eukaryotic type [1], [2].

Yeast Msk1 is a dual functional protein. In addition to aminoacylation of mitochondrial tRNA^Lys^ (tRK3), it has been proposed that Msk1 plays an essential role in the import of cytosolic tRNA^Lys^_CUU_ (tRK1) into mitochondria [3]. There are two other lysine isoacceptors in yeast cells: the non-imported nuclear encoded tRNA^Lys^_UUU_ (tRK2) and the mitochondrial DNA encoded tRNA^Lys^_UUU_ (tRK3) [4]. Further, the imported tRK1 that was specifically mutagenized to alter amino acid specificity was functional in yeast mitochondrial translation both *in vivo* and *in vitro* [5]. However, for tRK1 to be eligible for mitochondrial import, it has to go through a complex set of reactions that includes aminoacylation of tRK1 by cytosolic lysyl-tRNA synthetase, interaction of tRK1 with glycolytic enzyme enolase 2 (Eno2) and binding to the precursor form of Msk1 (pre-Msk1) [3], [6], [7]. The subsequent translocation across the mitochondrial membranes requires intact protein import machinery and ATP [3], [8]. The function of the imported tRNA is conditional and its import is also regulated by ubiquitin/26S proteosome [9], [10]. Further, the translocation across the mitochondrial membranes requires intact protein import machinery, ATP and additional un-identified cytosolic factors. However, the charged tRK1 imported into mitochondria by the pre-Msk1p mediated mechanism is only utilized in one cycle of translation as it cannot be re-charged by Msk1. The utilization of tRK1 in mitochondria is dependent on continuous action of cytosolic and mitochondrial tRNA synthetases. Hence the utilization of imported tRK1 is limited by the activity and availability of these two synthetases besides its function being restricted to one round of translation.

Yeast tRK1 can also be imported into human mitochondria in the presence of yeast cytosolic factors and Msk1 [5]. In addition, human cytosolic factors can replace yeast cytosolic factors in the presence of Msk1 to drive the import of tRK1 into human mitochondria [5]. It was previously suggested that human Kars might play a similar kind of role in the import of tRK1 into mitochondria [11]. However, there was no direct evidence showing that human Kars is indeed involved in the import of tRK1 into either human or yeast mitochondria.

We previously have shown that human mitochondrial and cytoplasmic lysyl tRNA-synthetases are expressed from alternative spliced mRNAs from a single gene [12]. We are interested to see whether human mitochondrial tRNA synthetase mitigates the role of yeast tRNA synthetases in the import and aminoacylation of tRK1 since the evolution and structural relatedness of these enzymes has been a subject of intense research for many years. Unlike yeast synthetases, both human cytosolic and mitochondrial lysyl-tRNA synthetases (Kars) are capable of aminoacylating yeast tRK1. Human mitochondrial Kars can substitute for yeast Msk1 for protein synthesis and tRNA import functions. Further, our findings suggest that human Kars facilitates the import of tRK1 into isolated yeast, rat and human mitochondria. In addition, human *KARS* partially suppresses the growth defects that are associated with yeast *MSK1* deletion. Interestingly, *in vivo* experiments suggest that import of tRK1 into yeast mitochondria is independent of yeast Msk1.

## Results

### Human mitochondrial lysyl-tRNA synthetase suppresses the growth defect associated with yeast *MSK1* deletion

Yeast cells deleted for *MSK1* do not grow on non-fermentable carbon sources as they are unable to aminoacylate mitochondrial tRNA^Lys^ (tRK3). This results in the inhibition of mitochondrial protein synthesis and loss of mtDNA. Before investigating the ability of human Kars to import tRK1 into yeast mitochondria, we determined whether Kars could substitute for the yeast Msk1 in the aminoacylation of tRK3. To test this, we introduced the human *KARS* cDNA or yeast *MSK1* on a high/low copy plasmids into a yeast strain heterozygously deleted for *MSK1* by insertion of a KANMX4 cassette (NS104). The resulting yeast strains were sporulated on fermentable and nonfermentable carbon containing media plates. In initial tests, all four meiotic progeny from diploid strains that contained high copy yeast *MSK1* formed colonies on both fermentable and non-fermentable carbon sources. Progeny from the diploid strain that contained high copy human *KARS* plasmid yielded four spores on fermentable and nonfermentable carbon sources. Out of the four spores, two were larger and wild type for *MSK1* gene while other two were smaller and positive for *MSK1* deletion (data not shown). Haploid progeny with *KARS* on a low copy plasmid failed to grow on non-fermentable carbon sources (data not shown). These results suggest that human *KARS* can substitute yeast *MSK1* deletion partially.

**Figure 1:**
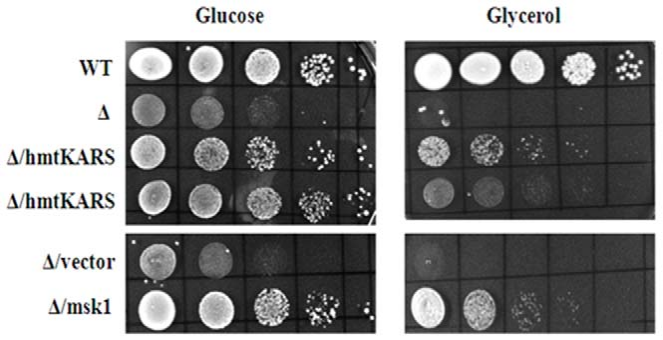
Suppression of growth defect associated with yeast *MSK1* deletion by human mitochondrial lysyl-tRNA synthetase. Strains carrying various ectopic plasmids were grown on YEPD medium over night. 2 0D_600_ unit cells were pelleted and suspended in 1 ml of water. The culture was serially diluted in 10-fold steps and 10 ul of each dilution was spotted onto YEPD and YEG plates. Two lanes of Δ/hmt*KARS* represent two spores originated from the single tetrad.

We investigated further the growth properties of the haploid progeny of strain NS104 containing high copy plasmids expressing human mitochondrial *KARS* or yeast *MSK1* under respiring and fermenting conditions. Cells were grown at 30°C in selective medium, serially diluted and spotted on rich carbon sources (YEPD) and on non-fermentable carbon sources (YEP plates supplemented with 3% glycerol/ethanol). Cells expressing high levels of human *KARS* partially suppressed the growth defect of *msk1*Δ cells on both fermentable and on non-fermentable carbon sources (Figure 1). The extent of suppression was comparable to that achieved by high levels of ectopically expressed *MSK1*. Cells deleted for *MSK1* exhibited poor growth on rich carbon sources compared to cells wild type for *MSK1* (NS101; Figure 1). The reason for the slow growth of the *msk1*Δ strain on rich carbon sources is not known, but it could be strain specific. However, loss of *MSK1* displayed a similar phenotype on non-fermentable carbon sources in other strain backgrounds ([3], present study). Further, human *KARS* poorly complements the growth defect at 37°C caused by *MSK1* deletion (unpublished results). This may be due to requirement of Msk1 at elevated temperature for the import of tRK1 [9].

Nevertheless, the results show that human *KARS* partially complements yeast *MSK1* deletion. We hypothesize that the human Kars can be imported into yeast mitochondria and substitute the function of yeast Msk1.

### Recombinant human mitochondrial lysyl-tRNA synthetase directs the import of tRK1 into yeast mitochondria

**Figure 2:**
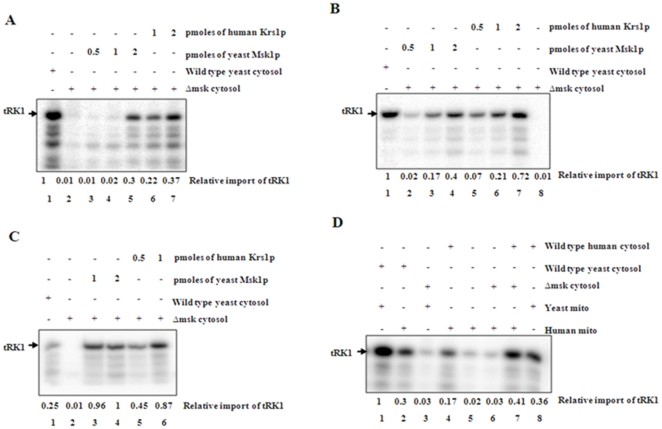
Import of tRK1 into yeast (Fig. 2A), rat liver (Fig. 2B) and human mitochondria (Fig. 2C) in the presence of human mitochondrial lysyl-tRNA synthetase. Import of tRK1 into isolated yeast or mammalian mitochondria was performed in the presence or absence (lane 2) of either yeast or human mitochondrial lysyl-tRNA synthetase (lanes 3–7). The import efficiency was assessed by RNase protection assay followed by polyacrylamide gel electrophoresis and phosphorimaging. Lane 1 contains wild type yeast cytosol that presumably contains both cytosolic and mitochondrial lysyl- tRNA synthetase. Fig. 2D. Substitution of yeast cytosol with human cytosol for the import of tRK1 into either yeast or mammalian mitochondria. Import of tRK1 was carried out into yeast (lanes 1, 2, 4 & 9) and human mitochondria (lanes 3, 5, 6, 7 & 8) in the presence of wild type yeast cytosol (lanes 2 & 3), or *mskΔ* cytosol (lanes 4, 7 & 8) or human cytosol (lanes 5, 8 & 9). The import efficiency was assessed by RNase protection assay followed by electrophoresis. The bands were quantified and relative tRK1 import values were mentioned by taking maximum imported sample as a value of 1.

We used an *in vitro* import system with bacterially expressed human Kars to investigate whether human Kars can import tRK1 into yeast mitochondria. Yeast mitochondria isolated from strain D273-10B [13] and were incubated with *in vitro* transcribed [^32^P]-labeled tRK1 under standard import conditions and the import efficiency was determined by RNase protection assay. tRK1 was imported into yeast mitochondria in the presence of wild-type yeast cytosol (Figure 2A, lane 1). Yeast cytosol is expected to contain both the cytosolic lysyl-tRNA synthetase and trace amounts of pre-Msk that are required for tRK1 import [3]. tRK1 was not imported into yeast mitochondria in the presence of a yeast cytosolic extract derived from a *msk1*Δ strain (Figure 2A, lane 2). However, the addition of purified human Kars (Figure 2A, lanes 6 and 7) or yeast Msk1 (Figure 2A, lanes 3–5) resulted in import of tRK1 into yeast mitochondria. A dose dependent increase in the import of tRK1 into yeast mitochondria was observed with increasing concentrations of added human Kars (Figure 2A, lanes 6 and 7). tRK1 was not imported into yeast mitochondria in the presence of yeast Msk1 or human Kars alone (data not shown). Similar results were obtained when yeast mitochondria substituted with mitochondria isolated from rat liver or human 143b cells (Figures 2B and 2C). These results show that human Kars can substitute for yeast Msk1 in targeting tRK1 into mitochondria *in vitro* in the presence of cytosol.

### Human cytosol can complement yeast cytosol in the import of tRK1 into yeast or mammalian mitochondria

Cytosolic extract from human cells was used to investigate whether human cytosol can complement the yeast cytosol in directing the import of tRK1 into mammalian and yeast mitochondria. Isolated mitochondria from human 143b cells, rat liver or yeast were incubated with ^32^P labeled ATP and processed as described in Methods. tRK1 was not imported into mitochondria in the absence of yeast or human cytosol (Figure 2D). Addition of mammalian cytosol to the reaction stimulated the import of tRK1 into human, rat liver or yeast mitochondria (Figure 2D). Cytosolic extract from a *MSK1* deletion strain failed to import the tRK1 either into yeast or mammalian mitochondria but the addition of purified human Kars or yeast Msk1 stimulated the import of tRK1 into mitochondria. These results show that human Kars or yeast Msk1 are equally efficient in stimulating the import of tRK1 and that human cytosol can substitute the role of yeast cytosol in directing the import of tRK1 into mitochondria *in vitro*.

### *In vivo* distribution of tRK1

Next, we analyzed the distribution of tRK1 in wild type and *MSK1* deletion strains. High resolution northern blot analysis suggested that more than 95% of the tRK1 is associated with cytoplasm and less than 5% is associated with mitochondria in wild-type cells (data not shown), consistent with previous studies [14]. Earlier studies showed the absence of tRK1 in the mitochondria of *msk1*Δ strain [3]. However, in our preliminary studies, a small but significant amount of tRK1 was associated with mitochondria in the *msk1*Δ strain (data not shown).

The association of tRK1 with mitochondria isolated from *msk1*Δ cells could be due to contamination of mitochondria preparation with cytosol. Loss of mitochondrial DNA as a result of *MSK1* deletion could change the mitochondrial morphology and result in enhanced association with the cytosolic fraction. However, we have observed decreased levels of mitochondrial-associated tRK1 in a rho^0^ strain that was generated by ethidium bromide treatment of parental strain NS101 (data not shown). To determine whether tRK1 was localized inside mitochondria or is a cytosolic contaminant, we used several methods that could eliminate cytosolic contamination with little reduction in the endogenous mitochondrial tRNA levels. First, we selectively permeabilized the mitochondrial membranes by digitonin detergent. Low concentrations of digitonin disrupt and partially solubilize the outer mitochondrial membrane as well as removing other membranous structures contaminating the mitochondrial fraction. To determine the concentration of digitonin that is required to reduce the cytosolic contamination, aliquots of mitochondria were treated with increasing concentrations of digitonin prior to centrifugation. Initially, the pellet and supernatant fractions were analyzed for the selective solubilization of specific proteins that serve as markers for the different mitochondrial subfractions. In subsequent experiments, nucleic acids were isolated from the digitonin soluble supernatant and insoluble pellet fractions and specific tRNA species were detected and quantitated by northern analysis.

**Figure 3:**
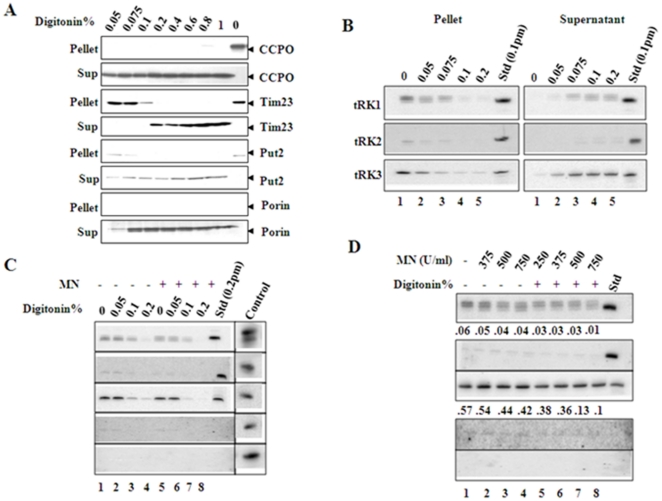
In vivo distribution of tRK1 in yeast cells. Isolated mitochondrial preparations were subjected to increasing concentrations of digitonin (Fig. 3A & B). Digitonin soluble (sup) and insoluble fractions (pellet) were separated by centrifugation. One set was used to analyze protein markers of outer membrane (porin), inter membrane space (CCPO), inner membrane (Tim 23) and the matrix (Put2) by SDS-PAGE followed by western blot (3A). In parallel, the other fraction was used to extract total RNAs and analyzed by northern blot for the presence of tRK1, tRK2 and tRK3 by using specific oligonucleotide probes. Isolated yeast mitochondrial preparations were treated with increasing concentration of digitonin from 0–0.2% (Fig. 3C) or 0.05% of digitonin (Fig. 3D) for 20 min on ice and reisolated the mitochondria by centrifugation. The pellet fraction was either treated with 250–750 units of MN (Fig. 3D) or 500 units of MN (Fig. 3C, lanes 5–8). Total RNA was extracted and analyzed by northern blot as above. Standard is the respective in vitro transcribed unlabeled tRNA that was used as a positive control except in the case of tCys and tPhe (Fig. 3C). Cytosolic fraction represents 2 µg of total RNA from the cytosol to show the levels of various tRNAs (Fig. 3C).

Treatment of mitochondria with 0.05% digitonin solubilized specifically the mitochondrial outer membrane, as shown by the release of the intermembrane space marker protein, CCPO into the supernatant (Figure 3A). Higher concentrations of digitonin solubilized the mitochondrial inner membrane, shown by the release into the supernatant of the matrix marker protein Put2 and the inner membrane protein Tim23 (Figure 3A). Endogenous tRK3 and imported tRK1 were released into the supernatant at the same concentrations of digitonin, which released the matrix protein Put2 (compare Figures 3A and 3B). These results indicate that mitochondrial membranes are intact and that the imported tRK1 fragments are found in the same intramitochondrial fraction as the endogenous mtDNA-encoded tRNA, tRK3. The levels of tRK2, a non-imported cytosolic tRNA, are reduced by 60–70% compared to the untreated mitochondria at 0.075% concentration (Figure 3B). It appears that digitonin resistant structures are still associated with nonspecific cytosolic tRNAs.

We determined if treatment of mitochondria with MNase (Micrococcal Nuclease) would reduce the contamination of cytosolic tRNAs. However, mitochondria treated with MNase alone showed contamination of cytosolic RNAs as evidenced by the presence of tRK2 (Figure 3D, lanes 2–4). We therefore developed alternative approach in which we treated mitochondria with increased concentrations of digitonin and fixed concentration of MNase or fixed concentration of digitonin and increasing concentrations of MNase to reduce non-specific association of cytosolic RNAs. It was previously shown that nuclear encoded small RNAs non-specifically associated with highly purified mitochondrial preparations but were sensitive to digitonin and MNase treatment [15].

In the first approach, mitochondria were treated with increasing concentrations of digitonin (Figure 3C, lanes 2–4) and then with a fixed concentration of MNase (Figure 3C, lanes 6–8). Aliquots of mitochondria were treated with increasing concentrations of digitonin (0–0.2%) for 15 minutes on ice. Mitochondria were re-isolated and treated with MNase at 500 U/ml for 25 minutes on ice and processed as described in the Methods. As shown in the figure 3C, low concentrations of digitonin (lanes 1–3) failed to reduce the cytosolic RNA contamination whereas the higher concentrations completely eliminates the cytosolic RNA levels but also reduces the endogenous tRNA levels significantly (Figure 3C, lane 4).

In another approach, aliquots of mitochondria were incubated with 0.05% digitonin to solubilize the outer membrane and then treated with different concentrations of MNase for 25 minutes on ice. Total nucleic acids were isolated and the presence of various tRNAs was detected by northern blot using specific probes. The contamination of tRK2 in samples treated with MNase alone was almost completely eliminated by treatment of the mitochondrial fraction with 0.05% digitonin and MNase (Figure 3D, lanes 5–8). This treatment had little effect on the amount of mitochondrial tRNA (represented by tRK3, Figure 3), even at the highest concentration of MNase.

The above experiments showed that neither digitonin nor MNase treatment alone was sufficient to remove the contaminating cytosolic tRNAs. However, we could reduce the amount of contaminating cytosolic RNAs by 97–99% without greatly reducing the endogenous RNA levels by a combined treatment of the mitochondrial fractions with digitonin and MNase. We used this combined treatment to further investigate import of tRK1 into yeast mitochondria.

### Import of tRK1 in yeast cells is independent of Msk1

**Figure 4:**
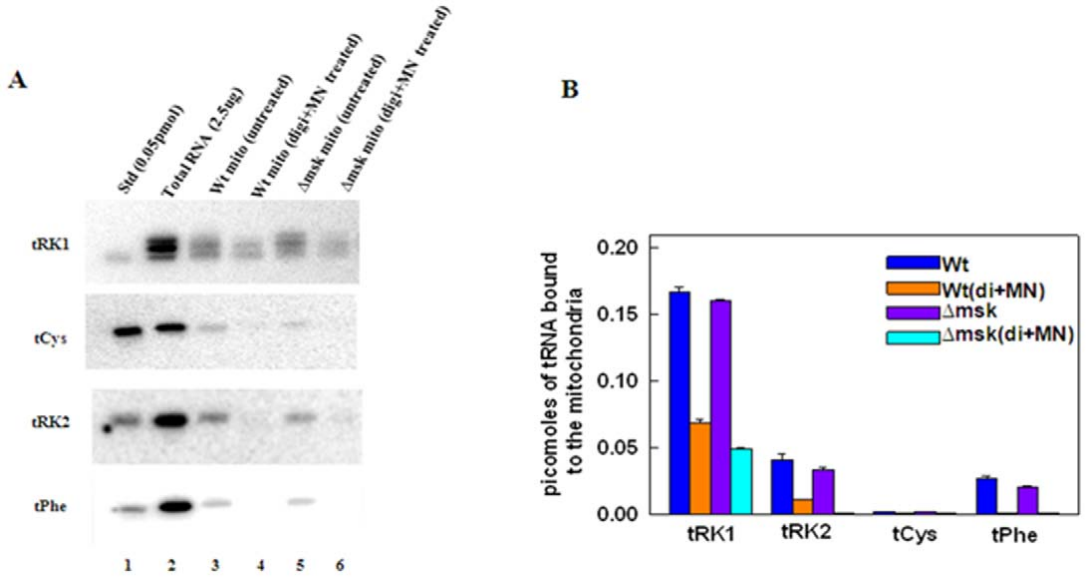
Presence of tRK1 in *msk1*Δ mitochondria. Mitochondria isolated from wild type or *msk1*Δ strains were treated with digitonin and MNase and total nucleic acids were separated on urea-acrylamide gel and analyzed by northern blot with respective probes. Total RNA represents the total cell RNA (2 µg) that was used to determine the levels of various tRNAs in the cytosol (Fig. 4A). Figure 4B represents the quantification of band intensities by densitometry.

To further investigate whether isolated mitochondria from *msk1*Δ strain contained any significant amounts of tRK1, we used the combined digitonin and MNase treatment described above to analyze the tRNAs associated with mitochondria. Mitochondria isolated from wild type and *msk1*Δ cells were treated with 0.05% digitonin for 30 minutes on ice, re-isolated, and treated with MNase at 500 U/ml for 25 minutes on ice. Total mitochondrial nucleic acids were then extracted and separated by urea-acrylamide gel electrophoresis and examined for the presence of representative cytosolic tRNAs (tRK1, tRK2, tRNA^Cys^, tRNA^Phe^) by northern analysis (Figure 4A). In the absence of digitonin and MNase treatment, as expected, a small portion of the cytosolic tRNAs are associated with mitochondria isolated from wild type and *msk1*Δ strains (Figure 4A, lanes 3 and 5). Treatment of mitochondria with digitonin and MNase reduced the level of most cytosolic tRNAs (tRK2, tRNA^Cys^, tRNA^Phe^). However, the amount of tRK1 is at least 10-fold greater than other cytosolic tRNAs, in both wild type and *msk1*Δ mitochondria (Figure 4A, lanes 4 and 6; Figure 4B). In mitochondria isolated from *msk1*Δ, the amount of tRK1 is decreased by 50% but its inaccessibility to digitonin and MN treatment (Figure 4A, lane 6) indicates that tRK1 is imported in these cells, despite the lack of Msk1. Results from three independent experiments suggest that tRK1 can be imported into mitochondria in the absence of Msk1 (Figure 4B).

**Figure 5:**
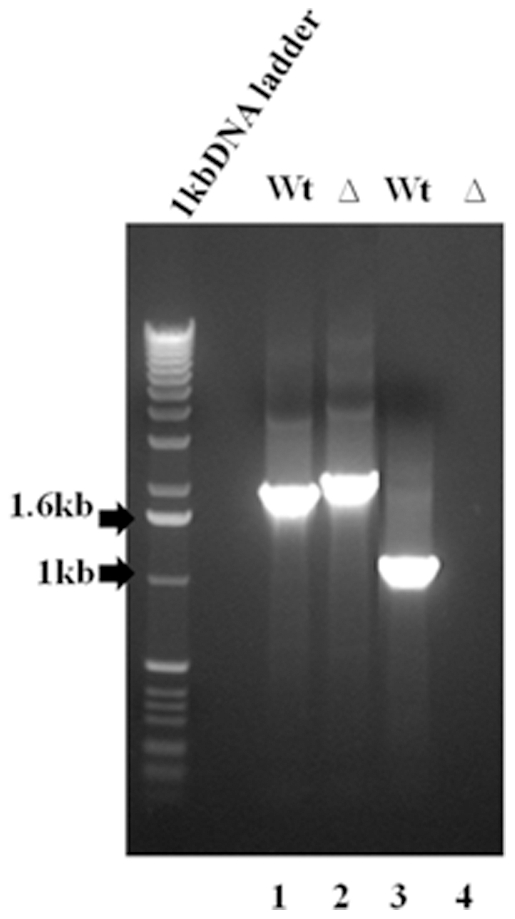
Confirmation of *MSK1* deletion in yeast strain by PCR analysis. Analytical PCR was performed with isolated genomic DNA from wild type and *msk1Δ* strain by using internal and upstream primers to detect the loss of *msk1* as mentioned in the Methods Section. Lane 1 represents the wt *MSK1* gene, lane 2 represents the KAN marker at *MSK1* locus, lane 3 represents the internal fragment generated by using internal primer in wild type and lane 4 represents the lack of *MSK1* gene in *msk1Δ* strain.

We excluded the possibility that the *MSK1* gene was not deleted or partially deleted in our commercially obtained *msk1*Δ strain, by PCR analysis. We isolated total genomic DNA from the parent and from the *msk1*Δ strain and performed PCR with primers flanking the open reading frame of *MSK1*. The putative *msk1*Δ strain contained the larger KanMX4 marker gene as the entire coding region of *MSK1* is replaced (Figure 5, lanes 1 and 2). We also used primers annealing to regions within the *MSK1* gene. As expected, *MSK1* gene product was obtained from the parent strain; no product was amplified from the *msk1*Δ strain (Figure 5, lanes 3 and 4). This analysis confirmed that *msk1*Δ cells indeed lacked the *MSK1* gene. We conclude that yeast mitochondrial lysyl-tRNA synthetase does not play a significant role in the import of native tRK1 into the mitochondrial matrix.

## Discussion

Yeast Msk1 is a dual functional protein, it aminoacylates the tRNA^Lys^ (tRK3) of mitochondria, and is also involved in the import of tRK1 into mitochondria [3]. We show here that human mitochondrial lysyl tRNA-synthetase (*KARS*) cDNA, when present in multiple copies, partially rescues the growth defect of *msk1*Δ cells on non-fermentable carbon sources. We also show that human Kars imports tRK1 into isolated yeast and mammalian mitochondria in the presence of yeast or human cytosolic factors. Our results provide genetic and biochemical evidence that human Kars can perform the role of yeast Msk1. The results presented here demonstrate for the first time that purified recombinant human Kars could complement the role of yeast Msk1 in the import tRK1 into isolated yeast mitochondria or into mammalian mitochondria *in vitro*.

It had been proposed that interaction between tRK1 and pre-Msk1 is absolutely essential for the import of tRK1 into mitochondria, since no tRK1 was detected in mitochondria from *msk1*Δ yeast cells [3]. However, we observe only slightly decreased levels of tRK1 in mitochondria from *msk1*Δ strain when compared to tRK1 levels in mitochondria isolated from a isogenic wild type strain. We show that association of tRK1 with mitochondria isolated from the *msk1*Δ strain is not due to non-specific contamination of cytosolic fraction. tRK1 is still detected in mitochondria isolated from *msk1*Δ strain and the mitochondria had been incubated with digitonin and MNase (Figure 3). It is clear from our results that the pre-Msk1 is not essential for import of tRK1 into mitochondria from the cytoplasm *in vivo*. Our results differ from a previous study [3] and this difference could be due to several reasons. The possibilities include a) a different strain background b) incomplete deletion of *MSK1* and c) different techniques to detect tRNAs. We employed high-resolution northern blot hybridization to detect mitochondrial tRNAs, whereas dot-blot hybridization method was used in previous studies which may not detect small quantities of tRNAs that are associated with mitochondria [3]. We also show that our *msk1*Δ strain lacks the *MSK1* gene by genomic PCR. However, we cannot rule out the possibility that the association of tRK1 in mitochondria isolated from *msk1*Δ strain is strain specific.

Our studies suggest that the *in vivo* import of tRK1 occurs independently of mitochondrial lysyl-tRNA synthetase. However, we observe that the import of tRK1 into mitochondria requires the presence of Msk1 *in vitro*. The *in vitro* import of tRK1 differs from other well-established systems in the requirement of many factors. Cytosolic factors are not required for the import of tRNAs into mitochondria of *Tetrahymena* [16], or in *Trypanosoma* [17] or in *Leishmania* [18] or in plants [19]. Nevertheless, it has been shown that aminoacylation is essential for the import of tRNA^Ala^ into *Arabidopsis thaliana* mitochondria. This evidence suggested that amino acyl-tRNA synthetases are indeed involved in the import of tRNAs into plant mitochondria [20]. However, the over-expression of *A. thaliana* alanyl-tRNA synthetase in yeast cells was not sufficient to import its cognate tRNA^Ala^ into the yeast mitochondria [21], indicating that the mechanism of import is different from plants to yeast. In yeast, aminoacylated tRK1 is essential for import, however, it is being utilized only once in the mitochondrial translation as Msk1 does not aminoacylate the cytosolic tRK1. The necessity of this conditional import of this tRNA into mitochondria could be a non-essential function of aminoacylated tRNA in mitochondrial translation as mitochondrial encoded tRK3 can decode both AAA and AAG codons. However, the imported aminoacylated tRK1 plays an important role at elevated temperature in decoding AAG codons as mitochondrial tRK3 is defective [9]. Recently, it has been shown that cytosolic Gln-tRNA is imported into yeast mitochondria and its import is independent of cytosolic factors and yeast mitochondrial Gln tRNA synthetase [22].

Cytosolic factors are essential as Msk1 is required for the import of tRK1 into isolated yeast mitochondria *in vitro*. The precise role of cytosolic factors in the import of tRK1 into mitochondria is not known. It was speculated that probably the cytosolic factors act to stabilize the pre-Msk/tRK1 complex or facilitate the binding of tRK1 complex to mitochondria [23]. Our *in vivo* studies show the existence of an import mechanism for the import of tRK1 that is not dependent on Msk1.

The import of precursor proteins or tRNA into mitochondria requires one or more outer membrane receptors [24]. It was observed that mitochondria can import preproteins with reduced efficiency when the cytosolic domains of the import receptors were removed by trypsin treatment. This residual import is called bypass import [25], [26]. It is possible that the import of tRK1 can be accomplished without the presence of Msk1 by alternative methods *in vivo*. In support of this hypothesis, we find reduced levels of tRK1 in mitochondria isolated from a *msk1*Δ strain when compared to the parental strain. We have also observed that cytosolic factors alone when present in large quantities, slightly stimulates the import of tRK1 into isolated yeast mitochondria in the absence of Msk1 *in vitro* (unpublished results). Our findings indicate that the *in vivo* import of tRK1 differs from the *in vitro* import. Our results also suggest that alternative import pathways are present for tRNA import and yeast can serve as a model system to study the evolutionarily divergent import pathways of tRNAs.

## Materials and Methods

### Yeast strains

BY4741 (*MAT*a/*MAT*α *his3Δ1 leu2Δ0 met15Δ0 ura3Δ0*), BY4742 (*MATα his3Δ1 leu2Δ0 lys2Δ0 ura3Δ*) and *msk1*Δ (*MAT*a *msk*::*KAN his3Δ1 leu2Δ0 met15Δ0 ura3Δ0*) strains were obtained from Research Genetics Inc. Heterozygous diploid strain NS104 (*MAT*a/*MAT*α *his3Δ1/his3Δ1 leu2Δ0/leu2Δ0 met15Δ/MET ura3Δ0/ura3Δ0 LYS/lys2Δ0*) was constructed by crossing strain BY4742 with *msk1*Δ. Strain NS108 is isogenic to NS104 but contains *MSK1* on a high copy plasmid (pTEF *MSK1* URA3-2μ). Strain NS112 (MATa *his3Δ1 leu2Δ0 met15Δ0 ura3Δ0*) contains human *KARS* on URA3-2μ plasmid. Standard yeast genetics and techniques were used [27].

### Plasmids and cloning

The complete coding sequence of human mitochondrial lysyl-tRNA synthetase (*KARS*) cDNA was amplified by PCR using Thermo polymerase (Ambion) with KARS-A primer (ACTAGTGAATTCATGTTGACGCAAGCTGCT) containing an EcoRI site and KARS-B primer (GGATCGATCTCGAGGACAGAAGTGCCAACTGT) containing a XhoI site. Human *KARS* and was cloned into the pGEM-T Easy vector to generate plasmid pNS33. pNS33 was digested with EcoRI and XhoI and the *KARS* ORF was isolated and cloned into a 2*μ* vector (*pTEF-URA3*) at EcoRI and XhoI sites [28] (a kind gift from Erica Johnson, TJU) to generate pNS37. *MSK1* was amplified using yeast genomic DNA as a template and primers MSK1-A (GGCACGTGACTAGTATGAATGTGCTGTTAAAA) and MSK1-B (GGATCGATAAGCTTTTACTGCCTGTTTACATC) and the PCR product was cloned into pGEM-T Easy vector to create pNS32. A SpeI/HindIII digestion product of pNS32 containing *MSK1* was inserted into *pTEF-URA* to generate pNS34. PCR was performed to check the insertion of KAN marker at *MSK1* locus with sense primer MSK1-C (TAGTCTTTTATTCGTGATAAAAGCGAAAAT) and antisense primer MSK1-D (CGTAGCGTAGTTTATTGGTGTAGAGAAAAA). To exclude the possibility of partial deletion of *MSK1* gene, PCR was performed with sense primer MSK1-C (TAGTCTTTTATTCGTGAAAAAAGCGAAAAT) and antisense primer MSK1-E (AATACGCAACTCCAAATGGTCGAAGTC CTT).

### Over expression and purification of human and yeast mitochondrial lysyl-tRNA synthetases

The human mitochondrial lysyl-tRNA synthetase cDNA cloned in pET 24d vector (Novagen) was expressed in *E. coli* BL21 (DE3) Codon Plus (RIL) cells and purified to 80% homogeneity as described [12]. The complete coding sequence of yeast mitochondrial lysyl-tRNA synthetase (*MSK1*) was PCR amplified and cloned into *E. coli* expression vector pET24d to generate a hexahistidine tag at the carboxyl terminal of synthetase. This construct was expressed in *E. coli* BL21 (DE3) Codon Plus (RIL) (Stratagene) at 15°C for 12 hours in the presence of 0.5 mM isopropyl-1-thio-β-D-galactopyranoside. The histidine tagged soluble pre-protein was partially purified using Talon metal affinity resin (Clontech) and concentrated. The purified Msk constituted approximately 70% of the total purified protein.

### Preparation of tRNA substrate and end labeling

A synthetic gene encoding tRK1 downstream from a T7 polymerase promoter was constructed by annealing ten overlapping oligonucleotides and cloning into EcoRI/BamHI digested pUC19 vector. This plasmid was linearized with Bst NI and used as a template for *in vitro* transcription using T7 polymerase. The tRK1 *in vitro* transcript was gel purified and stored in TE pH 8.0. tRK1 was labeled with α-[^32^P]-ATP at the 3′ end using *E. coli* terminal nucleotidyl transferase as described [29]. Prior to import assays, the tRNA was denatured at 80°C followed by slow cooling to 37°C in the presence of 15 mM MgCl_2_.

### Isolation of mitochondria and *in vitro* import assays

Mitochondria were isolated from yeast [30], rat liver [31] or from 143b osteosarcoma cell lines [29] as described. Yeast cytosolic factors were prepared as described [3]. 0.2 pmol of *in vitro* synthesized [^32^P]-labeled tRK1 was preincubated with 2 µg of yeast cytosolic factors with or without purified human Kars or yeast Msk1 on ice for 10 minutes prior to the import assay. Import of tRNA into mitochondria was performed in a volume of 100 µl containing 100 µg of mitochondria, 20 mM HEPES-KOH pH 7.2, 0.6 M sorbitol, 4 mM ATP, 1 mM GTP, 5 mM MgCl_2_, 25 mM KCl and 0.2 pmol of [^32^P]-labeled tRK1. The import reaction was carried out at 30°C for 25 minutes. Following import, mitochondria were re-isolated, suspended in import buffer and treated with 2.5 µg/ml RNases (Roche Biochemicals) for 30 minutes on ice to remove non-imported tRK1. Then mitochondria were reisolated and washed three times with SEM buffer (250 mM Sucrose, 10 mM MOPS pH 7.2, 1 mM EDTA). The washed mitochondrial pellet was resuspended in 0.1 M potassium acetate (pH-5.2), 0.5% (w/v) SDS and mitochondrial RNA was extracted using phenol-chloroform as described [3]. The total RNA was resolved on 12% urea-polyacrylamide gel electrophoresis and tRK1 was detected and quantitated by autoradiography or phosporimaging using a Molecular Dynamics PhosphorImager. Import of tRNA into isolated rat liver mitochondria or human cell lines were essentially performed as described [31]. In brief, isolated mitochondria were incubated with tRNA in the presence of energy mix at 30°C for 30 minutes and treated with RNases and processed as above. The Animal Ethics Committee of the University of Hyderabad approved the experiment protocol.

### Mitoplast Preparation

For generation of mitoplast, digitonin was used to permeabilize the outer mitochondrial membrane of mitochondria. Mitochondria were suspended at a concentration of 1 mg/ml in SEM buffer. 250 µg of mitochondria were incubated for 25 minutes on ice in 250 µl of SEM buffer containing 0 to 0.2% digitonin. The resulting mitoplasts were harvested by spinning at 14,000 rpm for 10 minutes and the supernatant was saved for further analysis. The mitochondrial pellet was washed once again with SEM buffer and was used either for micrococcal nuclease treatment or for the isolation of mitochondrial nucleic acids. To determine the efficiency of digitonin treatment, 100 µg of mitochondria were treated as above with digitonin, centrifuged, and the pellet and supernatant fractions separated by SDS-PAGE and transferred to a polyvinylidene difluoride membrane (Immobilon P, Millipore). Immunoblots were performed using antibodies specific for cytochrome *c* peroxidase (CCPO) (intermembrane space), TIM23 (inner membrane), delta-1-pyrroline-5-carboxylate dehydrogenase (Put2p) (matrix) and porin (outer membrane).

### Micrococcal Nuclease treatment of Mitochondria

For the Micrococcal nuclease (MNase) treatment, aliquots (250 µg) of mitochondria were resuspended in 2 mM CaCl_2_, 250 mM sucrose and 10 mM MOPS pH 7.1. MNase was added to 500 U/ml unless otherwise specified. MNase treatment was performed for 25 minutes on ice with occasional mixing. MNase was then inactivated by the addition of 10 mM EGTA on ice for 5 minutes. The mitochondrial suspension was diluted with SEM buffer containing 2 mM EGTA and centrifuged at 14000 g for 10 minutes. The reisolated mitochondrial pellet was washed one more time in SEM buffer. The resulting pellet was used to isolate total mitochondrial RNA.

### Northern blot

Mitochondrial nucleic acids were isolated from the various samples and were separated on 12% urea-acrylamide gels. The transfer of nucleic acids to the nylon membranes was performed as described [29]. Membranes were probed using T4 polynucleotide kinase-labeled oligonucleotide probes specific for tRK1, tRK2, tRK3, tRNA^cys^ and tRNA^Phe^. To detect tRK1, we used the oligonucleotide probe anti tRK1 (GTAGGGGGCTCGAACCCCTAACC) for tRK2, the probe anti tRK2 (GCCGAACGCTCTACCAACTCAGC) for tRK3, the probe anti tRK3 (TAGCTGGAGTTGAACCAAGCATG) for cytosolic tRNA^cys^, the probe anti tRNA^cys^ (TCAGGATCGAACTAAGGACCAAG) and for cytosolic tRNA^Phe^, the probe anti tRNA^Phe^ (TGAGAATCGAACTACATGTAAAT).
